# The art of war: using genetic insights to understand and harness radiation sensitivity in hematologic malignancies

**DOI:** 10.3389/fonc.2024.1478078

**Published:** 2025-03-21

**Authors:** N. Ari Wijetunga, Joachim Yahalom, Brandon S. Imber

**Affiliations:** ^1^ Department of Radiation Oncology, University of North Carolina, Chapel Hill, NC, United States; ^2^ Department of Radiation Oncology, Memorial Sloan Kettering Cancer Center, New York, NY, United States

**Keywords:** hematologic malignances, personalized medicine, radiation therapy, precision oncology, genomics, lymphoma

## Abstract

It is well established that hematologic malignancies are often considerably radiosensitive, which enables usage of far lower doses of therapeutic radiotherapy. This review summarizes the currently known genomic landscape of hematologic malignancies, particularly as it relates to radiosensitivity and the field of radiation oncology. By tracing the historical development of the modern understanding of radiosensitivity, we focus on the discovery and implications of pivotal mutated genes in hematologic malignancies such as *TP53, ATM,* and other genes critical to DNA repair pathways. These genetic insights have contributed significantly to the advancement of personalized medicine, aiming to enhance treatment precision and outcomes, and there is an opportunity to extend these insights to personalized radiotherapy. We explore the transition from early discoveries to the current efforts in integrating comprehensive genomic data into clinical practice. Specific examples from Hodgkin lymphoma, non-Hodgkin lymphoma, and plasma cell neoplasms illustrate how genetic mutations could influence radiosensitivity and impact subsequent radiotherapeutic response. Despite the advancements, challenges remain in translating these genetic insights into routine clinical practice, particularly due to the heterogeneity of alterations and the complex interactions within cancer signaling pathways. We emphasize the potential of radiogenomics to address these challenges by identifying genetic markers that predict radiotherapy response and toxicity, thereby refining treatment strategies. The need for robust decision support systems, standardized protocols, and ongoing education for healthcare providers is critical to the successful integration of genomic data into radiation therapy. As research continues to validate genetic markers and explore novel therapeutic combinations, the promise of personalized radiotherapy becomes increasingly attainable, offering the potential to significantly improve outcomes for patients with hematologic malignancies.

## Introduction

“Know your enemy and know yourself, and you can fight a hundred battles without disaster.”

-*The Art of War*, Sun Tzu

Oncologists must confront cancer at both the macroscopic level of the patient and the microscopic level of the cancer cell. The modern battle against cancer continues to rely on both systemic therapies, such as chemotherapy, immunotherapies and targeted agents, and local treatments, including surgery and radiation therapy (RT). While therapeutic decision making was historically driven largely by histology and stage, contemporary planning is increasingly reliant on molecular insights. Specifically, since the development of Sanger sequencing in the 1970s, scientists have meticulously mapped out somatic and germline DNA mutations that have already demonstrated strong potential to enhance and refine treatment approaches. In radiation oncology, however, there are currently no widely implemented examples of molecular traits determining appropriate RT utilization, RT targets and/or optimal RT doses. We now stand at a pivotal moment, with the opportunity to leverage this accumulated genetic intelligence to revolutionize our approach and take the offensive against cancer.

In this review, we will first describe the classical understanding of cancer radiosensitivity as it relates to DNA damage and the cell cycle. Next, we will review key studies that have identified specific genes which are related to radiation response. We will then discuss current efforts to develop genetic signatures of therapeutic sensitivity in hematologic malignancies, highlighting recent advancements and research findings. Lastly, we will comment on the potential future of personalized medicine within the field of RT for hematologic malignancies, highlighting potential developments and the implications for patient care.

## What is biologic radiosensitivity and radioresponsiveness?

“Attack him where he is unprepared, appear where you are not expected.”

-*The Art of War*, Sun Tzu

**Figure 1:**
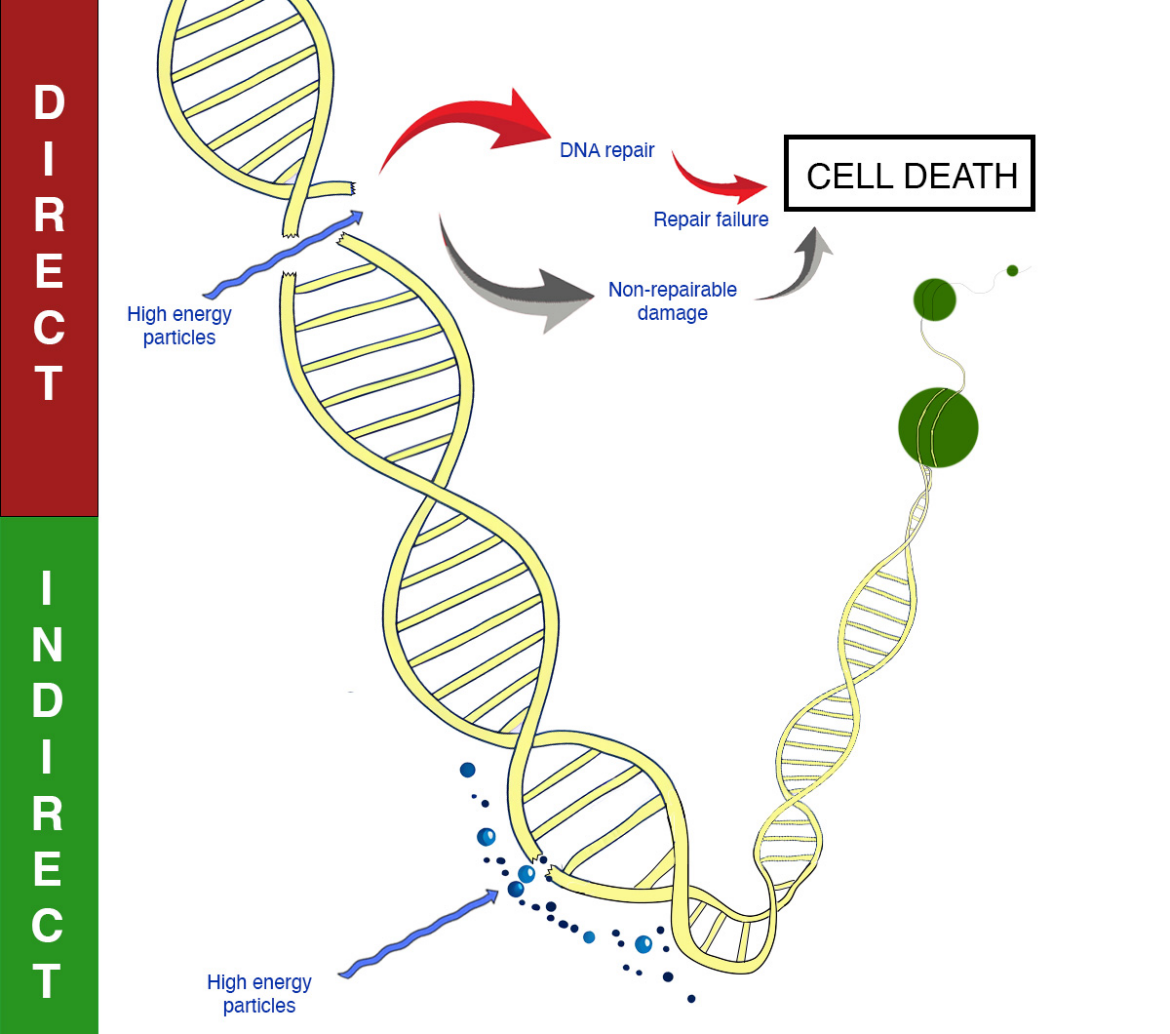
Mechanisms of DNA damage with radiotherapy. Direct and indirect DNA damage is shown.

RT exerts its lethal effects on cancer cells primarily through DNA damage. This damage is not uniformly distributed; it preferentially affects DNA in open chromatin regions compared to heterochromatin (1). Open chromatin is less densely packed and more transcriptionally active, making it more accessible to radiation-induced damage. Direct damage by radiation causes single-strand breaks (SSBs) and double-strand breaks (DSBs) in DNA. However, approximately two-thirds of the DNA damage caused by radiation is indirect, resulting from the generation of free radicals (**Figure 1**). These reactive oxygen species (ROS) are produced when radiation ionizes water molecules within the cell. The free radicals then diffuse through the cell, causing widespread damage to DNA, lipids, and proteins, resulting in base damage, SSBs, and DSBs. Hypoxic tumors, which have low oxygen levels, are therefore less susceptible to this indirect damage because the production of ROS is oxygen-dependent, leading to lower radiosensitivity (2).

“In war, the way is to avoid what is strong, and strike at what is weak.”

-*The Art of War*, Sun Tzu

The terms radiosensitive and radioresponsive are used in different contexts in clinical practice. They are sometimes referenced as a state of disease control in a clinical trial testing a radiotherapeutic intervention; for example, if the RT arm shows improved outcomes, patients treated in this study can be referred to as more radioresponsive. In a lesion-specific context, these terms may also reflect a local observation of a specific tumor decreasing in diameter, volume, or PET SUV following RT treatment. One may even use the term radiosensitivity to characterize the toxicity observed in a patient, referring to patients with more toxicity after RT as being more radiosensitive. However, in its most straightforward form, the concept of radiosensitivity can be defined by the observation that different phases of the cell cycle display varying levels of cell death in response to RT (3). For example, in the G0, early G1, and late S phases, cells are generally resistant to RT (4). Conversely, the most radiosensitive parts of the cell cycle are the late G1, G2, and M phases (4). Variability in radiation sensitivity throughout the cell cycle is thought to be related to the biological characteristics of each phase. For example, in the S phase, DNA synthesis leads to more nucleic acid content, a higher probability of DNA damage repair enzyme activity, and intrinsic free radical scavenging via glutathione (5). Tumor cells that proliferate at higher rates are generally seen as more radiosensitive, likely because a larger proportion of these cells are in the radiosensitive phases of the cell cycle. Hematologic malignancies can sometimes exhibit faster or more pronounced responses to relatively lower doses of radiation when compared to treated solid tumors, due to their rapid proliferation rates and the characteristics of the cells in these malignancies (6). To understand the observed inherent radiosensitivity of hematologic malignancies, one must first understand how DNA damage caused by radiation is repaired.

## How do cells detect and repair DNA damage?

“What is of supreme importance in war is to attack the enemy’s strategy”

-*The Art of War*, Sun Tzu

**Figure 2:**
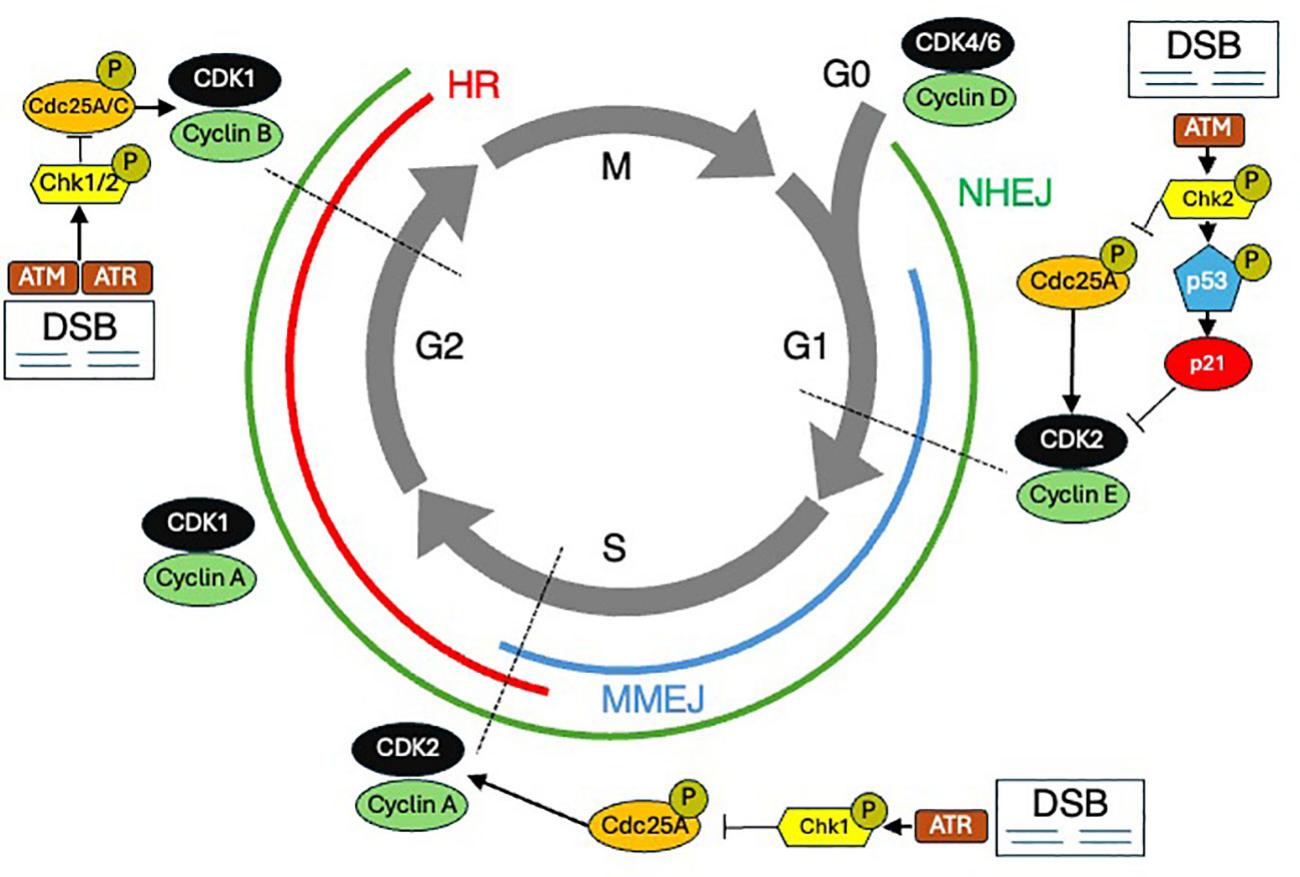
The cell cycle and DNA damage repair (DDR). The cell cycle is shown with various DDR mechanisms shown at the stages during which they predominate. DSB Double strand breaks. HR Homologous recombination. MMEJ Microhomology-mediated end joining. NHEJ Nonhomologous end joining.

Cells have evolved sophisticated strategies to halt the cell cycle and repair DNA damage, and cancer cells have developed methods to overcome these checks and balances (**Figure 2**). Normal cells have DNA damage repair mechanisms that maintain genomic integrity, involving various pathways to address different types of DNA damage. The MRN complex, consisting of MRE11, RAD50, and NBS1, plays a critical role in detecting and signaling DSBs (7). Upon recognizing damage, the MRN complex recruits ATM (ataxia-telangiectasia mutated kinase), which phosphorylates several key proteins, including p53 and H2AX, to initiate the DNA damage response (DDR). There are two major pathways to repair DSBs: homologous recombination (HR) and non-homologous end joining (NHEJ). HR, active during the S and G2 phases of the cell cycle, uses a sister chromatid as a template for accurate repair, involving proteins such as BRCA1, BRCA2, and RAD51. In contrast, NHEJ, which operates throughout the cell cycle, directly ligates the broken DNA ends but is more error-prone. NHEJ has core components including Ku70/80, DNA protein kinases, LIG4, XRCC4, and XLF. In addition to DSB repair, cells employ mismatch repair (MMR) to correct replication errors, nucleotide excision repair (NER) to remove bulky DNA adducts caused by UV radiation, and base excision repair (BER) to fix small base lesions induced by oxidative stress. The coordination of these pathways and others ensures comprehensive maintenance of DNA integrity and is crucial for preventing mutations that could lead to cancer.

Relative resistance to radiation-induced damage in the S phase is thought to be due in part to an elevated amount of DNA synthesis and repair enzymes, as well as increased intracellular levels of glutathione (a free radical scavenger) (8). Cell cycle blockage in the G1 phase after ionizing radiation is believed to allow time for the recognition and repair of DNA damage prior to the initiation of DNA synthesis. Cells are most sensitive in the G2/M phase of the cell cycle, possibly because there is no time for adequate repair before chromosome segregation takes place. Several gene products have been identified which increase expression or are post-translationally altered following DNA damage, and these are thought to participate in halting cell cycle progression (9). These damage-responsive genes and their protein products include p53, p21, growth arrest and damage-delay (GADD45), X-ray induced protein (XIP269), retinoblastoma protein (Rb), and a group of retinoblastoma control proteins (RCPs), which bind to Sp1 sites in DNA promoters and may act to further alter gene transcription in response to DNA damage (10). Understanding the mechanisms that underlie radiation effects on DNA and the cell’s ability to counter these effects highlights that tumors with highly deregulated genomes will exhibit abnormal cell cycle biology, a potentially advantageous observation for inducing radiosensitivity.

## Historical perspectives on genetic associations with radiosensitivity

“In war, the victorious strategist only seeks battle after the victory has been won, whereas he who is destined to defeat first fights and afterwards looks for victory.”

-*The Art of War*, Sun Tzu

A rational starting point to identify potential somatic molecular alterations in cancer cells which might be associated with increased radiosensitivity is to review early key observations linking individual genetic mutations to syndromes characterized by heightened radiation toxicity. These putative genes are summarized in **Table 1**. One of the first significant discoveries was the identification of the *ATM* gene, which is crucial in Ataxia-Telangiectasia, a disorder marked by increased sensitivity to ionizing radiation (14, 15). Similarly, mutations in the *NBS1* gene, part of the MRN complex, cause Nijmegen Breakage Syndrome (NBS), another autosomal recessive disorder with increased radiosensitivity and cancer predisposition (16). The discovery of the *TP53* gene, often referred to as the “guardian of the genome,” highlighted its critical role in the cellular response to DNA damage (17–19). Li-Fraumeni Syndrome, caused by germline mutations in *TP53*, underscored the impact of genetic mutations on radiosensitivity (20). Additionally, Xeroderma Pigmentosum (XP) is marked by extreme sensitivity to UV radiation due to defects in the NER pathway (21), while Fanconi Anemia (FA), characterized by mutations in *FANCA* and other FA genes, increases susceptibility to DNA crosslinking agents and radiation, demonstrating the role of DNA repair pathways in maintaining genomic stability (22).

**Table 1:** Summary of major DNA damage repair genes, associated syndromes, prevalence, known targeted agents, and associated cancers (11–13).

Gene symbol	Full gene name	Associated syndromes	Prevalence	Type of DNA damage sensing/repair disrupted	Known drugs targeting	Associated cancers
XRCC1	X-ray Repair Cross Complementing 1	No specific syndrome, associated with increased cancer risk	Rare, exact prevalence unknown	BER	No specific drugs, but associated with response to platinum-based therapies	Multiple cancers including Breast, Lung, Cervical
CHK2	Checkpoint Kinase 2	Li-Fraumeni-like Syndrome	Rare, exact prevalence unknown	DDR, cell cycle checkpoint control	CHK2 inhibitors (e.g., CCT241533)	Breast, Lung, Lymphoid malignancies
TP53	Tumor Protein p53	Li-Fraumeni Syndrome	< 1 in 20,000	DDR, cell cycle checkpoint control, apoptosis	MDM2 inhibitors (e.g., RG7112, Nutlin-3)	Multiple cancers including Breast, Sarcomas, Leukemia
MRE11	MRE11 Homolog	Ataxia-Telangiectasia-Like Disorder (ATLD)	Rare, exact prevalence unknown	DSB	No specific drugs, but crucial in DNA damage signaling and repair	Lymphoid malignancies
RAD50	RAD50 Double Strand Break Repair Protein	Nijmegen Breakage Syndrome	1 in 100,000	DSB	No specific drugs, but associated with DDR pathways	Lymphoid malignancies
ATM	Ataxia-Telangiectasia Mutated	Ataxia-Telangiectasia (A-T)	< 1 in 100,000	DSB, DNA damage signaling	ATM inhibitors (e.g., AZD0156, AZD1390, KU-55933)	Lymphoid malignancies, Breast, Prostate
NBS1	Nijmegen Breakage Syndrome 1	Nijmegen Breakage Syndrome	1 in 100,000	DSB, DNA damage signaling	None currently approved	Lymphoid malignancies
WRN	Werner Syndrome RecQ Helicase-Like	Werner Syndrome (Adult Progeria)	Rare, exact prevalence unknown	HR, DNA replication, Telomere maintenance	No specific drugs, but potential targets in aging and cancer	Sarcomas, Skin cancer, Rare leukemias
BRCA2	Breast Cancer 2	Hereditary Breast and Ovarian Cancer (HBOC)	1 in 400 to 1 in 800	HR	PARP inhibitors (e.g., Olaparib, Rucaparib)	Breast, Ovarian, Prostate
BRCA1	Breast Cancer 1	Hereditary Breast and Ovarian Cancer (HBOC)	1 in 400 to 1 in 800	HR	PARP inhibitors (e.g., Olaparib, Rucaparib)	Breast, Ovarian, Prostate
RAD51	RAD51 Recombinase	No specific syndrome, associated with increased cancer risk	Rare, exact prevalence unknown	HR	No specific drugs, but RAD51 inhibitors in development	Breast, Ovarian, Prostate
FANCD2	Fanconi Anemia Complementation Group D2	Fanconi Anemia	1 in 100,000 to 1 in 350,000	Interstrand Crosslink Repair, HR	No specific drugs, but sensitivity to DNA crosslinking agents	Acute Myeloid Leukemia, Squamous Cell Carcinoma
FANCA	Fanconi Anemia Complementation Group A	Fanconi Anemia	1 in 100,000 to 1 in 350,000	Interstrand crosslink repair, HR	No specific drugs, but sensitivity to DNA crosslinking agents	Acute Myeloid Leukemia, Squamous Cell Carcinoma
MSH2	MutS Homolog 2	Lynch Syndrome	1 in 500 to 1 in 3,000	MMR	No specific drugs, but sensitivity to immunotherapy and chemotherapies	Colorectal, Endometrial, Ovarian
MLH1	MutL Homolog 1	Lynch Syndrome	1 in 500 to 1 in 3,000	MMR	No specific drugs, but sensitivity to immunotherapy and chemotherapies	Colorectal, Endometrial, Ovarian
MSH3	MutS Homolog 3	Lynch Syndrome (less common)	Rare, exact prevalence unknown	MMR	No specific drugs, but relevance in combination with other MMR genes	Colorectal, Endometrial, Ovarian
ERCC1	Excision Repair Cross-Complementation Group 1	Xeroderma Pigmentosum (with other NER genes)	1 in 1,000,000	NER	No specific drugs, but associated with sensitivity to platinum-based chemotherapy	Skin cancer, Lung cancer
PARP1	Poly (ADP-Ribose) Polymerase 1	No specific syndrome	Rare, exact prevalence unknown	SSB, BER	PARP inhibitors (e.g., Olaparib, Rucaparib)	Breast, Ovarian, Prostate

DDR DNA damage response, BER base excision repair, HR homologous recombination, MMR mismatch repair, NER nucleotide excision repair, DSB double strand breaks, SSB single strand breaks.

In addition to syndromes that were directly linked to radiation sensitivity by a single altered gene, other DNA damage sensing and repair gene alterations are also associated with increased risk of cancer and radiation sensitivity, though with reduced penetrance. The identification of mutations in the *BRCA1* and *BRCA2* genes, pivotal in HR repair of DSBs, was associated with a breast cancer risk as high as 70% and emphasized the importance of intact DDR in mitigating cancer risk (23, 24). RAD51 is an essential component for fixing radiation-induced DSBs as part of HR, and germline *RAD51* mutations are associated with a 10-20 percent lifetime risk in women for ovarian, fallopian tube, or primary peritoneal cancer and a 30% risk for breast cancer (25). Alterations in *XRCC1*, involved in the BER pathway crucial for repairing SSBs induced by ionizing radiation, have been associated with increased risk for several cancers (26). Similarly, the elucidation of the *CHK2* gene as a key checkpoint kinase in DDR, particularly to ionizing radiation, marked another important discovery. The presence of *CHK2* mutations can double the lifetime risk of breast cancer and increase colorectal and prostate cancer risks (27–29). Each of these genetic insights has deepened our understanding of DNA repair mechanisms, and as RT exerts its effect on cells through DNA damage, the presence of germline and somatic DDR alterations has implications for both cancer risk and radiosensitivity. For example, identifying a single altered copy of a cancer-associated gene has allowed clinicians to begin augmenting cancer treatments through exploiting synthetic lethality (30), and agents are already being used to increase radiosensitivity of cells with impaired DDR (31).

Given the effects that radiation has on normal and cancer cells, it is not surprising that cell signaling and transcription factor pathways have also been increasingly implicated in mitigating aspects of radiation sensitivity. The JAK-STAT pathway allows extracellular signals including cytokines such as interferons and growth factors to quickly influence nuclear processes. Many types of cancer have shown that STAT3 can mediate resistance to chemoradioimmunotherapy (32), and targeting STAT3 may overcome radioresistance (33). The Notch pathway involves 4 short-range cell-cell signaling receptors regulating genes involved in cell cycle regulation, cellular differentiation, and stem cell maintenance. Of note, NOTCH1 inhibits the kinase activity of ATM, and blocking Notch in the presence of DNA damage leads to increased radiation sensitivity in an ATM-dependent manner (34). Additionally, inactivation of HR in Notch-driven cancers is shown to cause radiosensitization (35). The Nuclear factor (NF)-κB transcription factor regulates immunity, cellular survival and apoptosis. DSBs, like those resulting from radiation therapy, activate the NF-κB pathway (36). Many other cell signaling pathways are implicated in radiation sensitivity and are active areas of research. They may yield mechanistic insight into radiation response when they are altered or may be targeted to induce radiation sensitivity.

While there are no guideline-approved variations in radiation therapy indications based on clinicogenetic factors, recent insights from solid tumors offer promising directions. For instance, solid tumors with *ATM* or *BRCA* mutations exhibit increased radiosensitivity compared to matched controls (37, 38). Similarly, the radiosensitivity of human papillomavirus (HPV)-associated oropharyngeal cancer (OPC) is partly attributed to deficient DNA repair caused by E6 and E7 viral oncoproteins, which degrade p53 and inactivate Rb, disrupting DNA repair pathways and enhancing susceptibility to radiation-induced DSBs (39, 40). This intrinsic radiosensitivity of HPV-positive OPC has facilitated dose de-escalation studies, demonstrating that reduced radiation doses (e.g., 30-60 Gy versus 70 Gy) can achieve comparable local control while minimizing treatment-related toxicity (41). Furthermore, HPV-positive tumors are less likely to harbor hypoxic microenvironments—an important determinant of radioresistance—further enhancing their radiation responsiveness (42). These findings underscore opportunities for tailored radiation dosing and emphasize the importance of considering tumor microenvironmental factors when optimizing radiotherapy for other cancers. Lastly, the Oncotype DX DCIS score was retrospectively correlated with local recurrence risk after lumpectomy for DCIS, and it can guide the use of adjuvant RT (43). However, there are no large-scale prospective trials randomizing DCIS patients to omit radiation solely using this score, and it is primarily used in risk-stratification and shared decision-making contexts with questionable clinical utility and cost-effectiveness (44).

## Can personalized medicine reduce radiation-associated toxicities?

“He who wishes to fight must first count the cost.”

-*The Art of War*, Sun Tzu

Radiation therapy is a powerful tool in cancer treatment, but no therapy comes without a price: adequate radiation dose to effectively treat a tumor and radiation-associated toxicity to surrounding normal tissues are two sides of the same coin. As discussed above, there are several genetic variants especially those involved with DDR that have been associated with augmented toxicity to RT; this emphasizes a potential to identify patients who may be less able to adequately repair DNA damage to normal cells (45). However, the results of studies trying to associate DDR genes and cell cycle genetic aberration to radiation toxicity are not always straightforward. For example, in a proof-of-principle study, the *ATM* gene was sequenced in 20 patients with severe late radiation side effects, but no *ATM* mutations were found (46). Since that time, there has been inconsistent data in gene-level and population-based studies, though, more recently, *ATM* sequence variants were shown to predict adverse RT response in prostate cancer patients (47, 48). Alternative mechanisms of radiation toxicity are also implicated such as nucleoshuttling of ATM (49). A recent study introduced the PROSTOX assay, a microRNA-based test that may be able predict the risk of long-term genitourinary toxicity in prostate cancer patients undergoing radiation therapy by looking for specific germline microRNA single-nucleotide polymorphisms (SNPs) (50). SNPs and *TP53* polymorphisms correlate with severe late adverse effects and clinical outcomes in cancer patients undergoing RT (51, 52). Specifically, certain *TP53* mutations predict normal tissue toxicity following RT in head and neck cancer (53). Similarly, the presence of *BRCA1*/*BRCA2* mutations in breast cancer patients may influence the risk of complications after radiation, like brachial plexopathy, though the data is conflicting (54, 55). The Radiosensitivity Index (RSI) predicts a tumor’s response to radiation by analyzing a 10-gene signature related to DDR and cell cycle regulation (56), with specific studies showing its utility in reducing breast cancer treatment toxicity (57). Building on RSI, a dose-adjustment algorithm termed GARD (genomic adjusted radiation dose) assayed multiple solid tumors and integrated the radiosensitivity score with RT dose, optimizing therapeutic outcomes while significantly minimizing the risk of radiation-induced toxicities (58). GARD was associated with risk of local recurrence in breast cancer and has the potential to be used to make decisions on radiation dose adjustment (59). Therefore, it is possible that a general test such as RSI and GARD, or a novel disease-specific genetic signature may be used to optimize radiation doses through personalized escalation or de-escalation.

## Genomics of hematologic malignancies and potential prediction of treatment response

“In the midst of chaos, there is also opportunity.”

-*The Art of War*, Sun Tzu

Decades of research into molecular biomarkers has yielded an undeniable truth: the genome is incredibly intricate and its complexity is often difficult to translate into clinically relevant information. We are no longer in an era where single genes establish new syndromes with easily observable phenotypes. While certain single nucleotide variants (SNVs) are associated with targeted therapies (60), the associations with radiosensitivity are more complicated, often involving defects in multiple genes or entire pathways. This complexity necessitates a more nuanced understanding of the mutational landscape and its impact on therapeutic sensitivity.

Preclinical models such as cell lines and transgenic mice have contributed our earliest observations about radiation sensitivity in hematologic malignancies, indicating that lymphomas are at one extreme of the spectrum of radiosensitivity relative to solid tumors like melanoma and glioblastoma (61, 62). In Mantle cell lymphoma (MCL), cell lines derived from patients indicated that observed radiosensitivity may be due to distinct mechanisms in subtypes of MCL such as telomere shortening, loss of heterozygosity at the *ATM* locus, and the functionality of *TP53* mutants (63). Paradoxically, in mice with lymphomas arising in a background of altered *ATM*, *TP53*, *ARF*, or *NBS1*, showed that mice with an intact DDR had the most durable remission after irradiation (64). In Burkitt lymphoma (BL) cell lines, mutant *TP53* abrogated the ability of G-phase arrest following radiation (65). Additional studies of lymphoma cell lines have shown that in the presence of wild type *TP53*, having mutations in downstream proteins can result in similar radiosensitivity (66). Using exogenous agents to inhibit NF-KB signaling has been shown to radiosensitize BL cell lines (67). Studies of mouse lymphoma cell lines show that radiation resistance correlates to Bcl-2 expression and suggest that Bcl-2 blocks apoptosis by the antioxidant pathway (68). The transition from candidate gene studies to genome-wide association studies (GWAS) in identifying genetic markers of RT toxicity and response will likely involve genomic profiling (69). Through target sequencing panels, whole exome sequencing (WES), and whole genome sequencing (WGS) approaches, a vast amount of molecular information can inform clinical radiation oncology practice. Studies that establish models in which genes are associated with radiation sensitivity or predict patient response could be translated into a framework for personalized RT (70).

**Figure 3:**
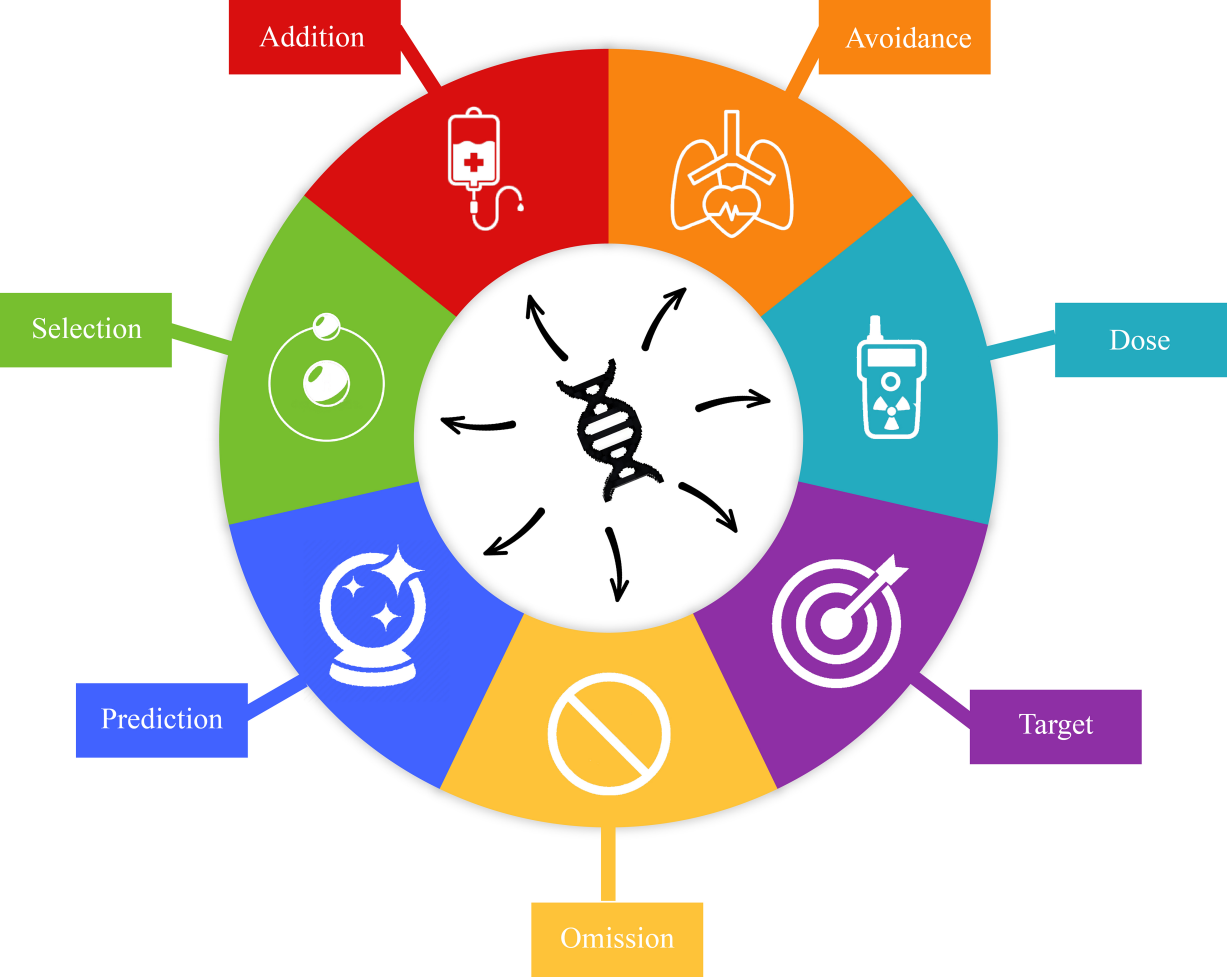
The potential uses of genetic information in radiotherapy (RT). Addition addresses the identification of mutations such as those implicated in synthetic lethality that may be targeted and used in conjunction with RT. Avoidance concerns the germline mutations that may indicate that a patient has greater radiosensitivity of their normal tissue. Dose can be reduced or escalated depending on observed genetic phenotypes. The potential for tumor genetics to provide insight into the behavior of different lesions within the same patient or the aggressiveness of a particular lesion has implications for targeting radiation (*i.e.*, only targeting the largest lesion out of several). Tumor genetics may indicate that RT is not the preferential mode of treatment and provide insight into omission of RT. Genetics can be used to predict the likelihood of RT eradicating the tumor. Lastly, the RT modality (*i.e.* photons, protons, brachytherapy, etc.) may be chosen by understanding the tumor genetics.

A thorough understanding of the genetic underpinnings of hematologic malignancies have already led to advancements in targeted systemic therapy options. For example, Tazemetostat inhibits the EZH2 protein, frequently altered in lymphomas, and phase II trials have demonstrated benefit in patients with *EZH2* mutations (71). Moving forward, we foresee that these same insights could facilitate personalized consideration and integration of RT. Specifically, these opportunities include a better understanding of how these aforementioned targeted therapies could be optimally used in conjunction with RT. Furthermore, these insights may highlight opportunities for rational RT dose alterations or even RT inclusion/omission, treatment-response prediction, and RT modality selection (**Figure 3**). There may also be opportunity to better predict risk of normal tissue toxicity post-RT.

In the following sections, we will review the current landscape of common genetic mutations in hematologic malignancies, focusing on their implications for treatment sensitivity and resistance.

## Hodgkin lymphoma

Hodgkin lymphoma (HL) is challenging to characterize from a genomic perspective given that the main neoplastic cells, Hodgkin and Reed-Sternberg (HRS) cells, comprise a minority of the tumor microenvironment (<5%). Only recently has it become realistic to isolate HRS cells for better characterization; whereas, most existing work relied on bulk sequencing with admixed cell populations. The observed mutations in HL are shown in **Table 2**. From targeted sequencing panels of FFPE biopsy samples, TP53 is the most frequently mutated gene in HL (approximately 20% of patients) (78). BCL2, an antiapoptotic factor, is thought to be involved in the pathogenesis of HL, and overexpression of BCL2 in HL is correlated with poorer response to chemotherapy (79). Microdissected HL samples indicated that EP300 and CREBBP, epigenetic regulators, are mutated in up to 40% of samples (80). No studies have identified specific genetic markers that can determine the radiosensitivity of HL; however, genetic aberrations are found in the immune system, JAK-STAT, NF-κB, DNA repair and cell cycle pathways, indicating that there are potentially radiosensitive HLs depending on the underlying molecular characteristics (81).

**Table 2:** Summary of Hodgkin lymphoma and associated genetic aberration (72–77).

Hodgkin lymphoma type	Known subtypes and prevalence	Major mutated genes and prevalence	Known CNAs	Known translocations	Differentially expressed genes (RNA/Protein)
Nodular Sclerosing	Subtypes: NS1, NS2; Prevalence: 60-80%	*B2M* (40-50%), *SOCS1* (30-40%), *GNA13* (20-30%)	9p24.1 amplification, 2p16.1 gain, 6q deletion	Rare	CD30 (IHC), CD15 (IHC), PDL1/PDL2 (IHC); SPARC, CTSK, COLI
Lymphocyte-Depleted	1-2%	*TP53* (20-30%), *SOCS1* (30-40%), *B2M* (10-15%)	9p24.1 amplification, 7q deletion, 17p loss	Rare	CD30 (IHC), PDL1/PDL2 (IHC), MUM1 (IHC)
Lymphocyte-Rich	5-10%	*SOCS1* (30-40%), *B2M* (20-30%), *TNFAIP3* (10-20%)	2p16.1 gain, 6q deletion	Rare	CD30 (IHC), CD15 (IHC), PDL1/PDL2 (IHC), OCT.1 (IHC), OCT.2 (IHC), BOB.1 (IHC), BCL6 (IHC)
Mixed Cellularity	15-30%	*B2M* (40-50%), *TNFAIP3* (20-30%), *STAT6* (15-20%)	9p24.1 amplification, 6q deletion, 17p loss	Rare	CD30 (IHC), PDL1/PDL2 (IHC), CD15 (IHC); 1Qα, C1Qβ, CXCL9
Nodular Lymphocyte-Predominant	5-10%	*TNFRSF14* (50-60%), *BCL6* (20-30%), *JUNB* (15-25%)	9p24.1 gain, 2p16.1 gain	t(2;5)(p23;q35) - *NPM1*-*ALK* in rare cases	CD20 (IHC), BCL6 (IHC), CD45 (IHC), OCT.2 (IHC), BOB1 (IHC), OCT.1 (IHC) Pax-5 (IHC), KLHL6 (IHC), GCTE-1 (IHC)

Though *ATM* is not mutated at high frequencies in HL, ataxia-telangiectasia and Rad3-related (*ATR*), which is also essential for proliferation, is shown to be mutated in some HL cell lines (82). Previous studies suggest that ATR might participate in the signaling of ionizing radiation (IR)- and ultraviolet (UV)-induced DNA damage (83). ATR is activated not only by UV-induced SSBs but also other forms of DNA damage and replication blocks, with mutations believed to cause abrogation of ATR in HL (82). Other cell cycle genes like *KLHDC8B61 (* 84 *)* are *NPAT (* 85 *)* (nuclear protein, ataxia-telangiectasia), are also implicated in HL, and may convey similar radiosensitivity to *ATM*/*ATR* mutations. Finally, mutations in *POT1* in HL are associated with increased chromosomal instability (CIN) which may associate with increased radiosensitivity (86). Copy number alterations (CNA), which involve the gain or loss of DNA segments directly affecting gene dosage, are present in more than 20% of HL cases and are enriched in genes related to NF-κB signaling, such as *REL, IKBKB, CD40*, and *MAP3K14 (* 87 *)*. The presence of specific recurrent CNAs in HL was shown to be related to chemotherapy resistance (88).

## Non-Hodgkin lymphoma and other lymphoid malignancies

**Figure 4:**
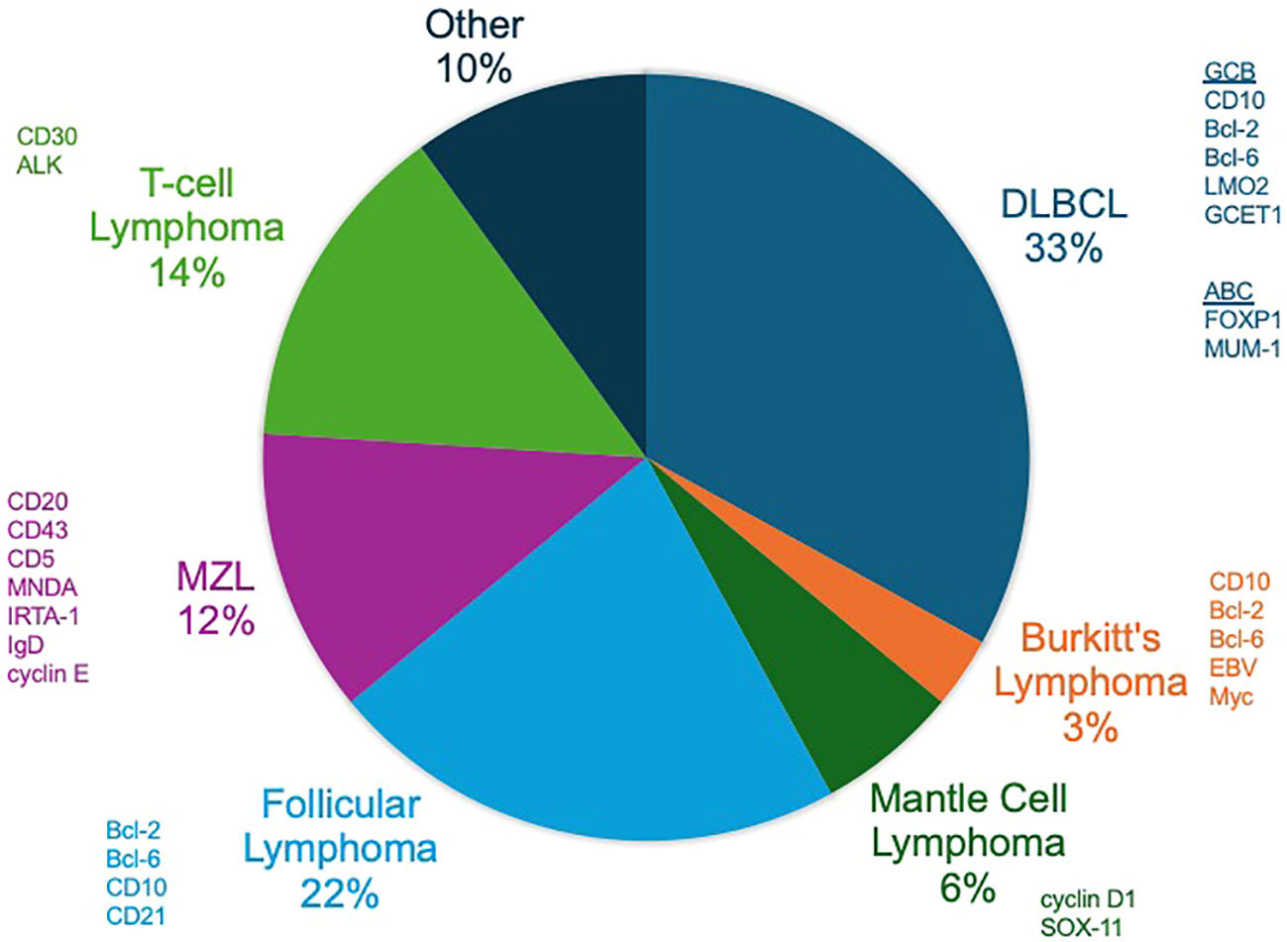
Non-Hodgkin lymphomas (NHLs). The distribution of the prevalence of the most common NHLs with commonly expressed factors on histopathology shown.

**Figure 5:**
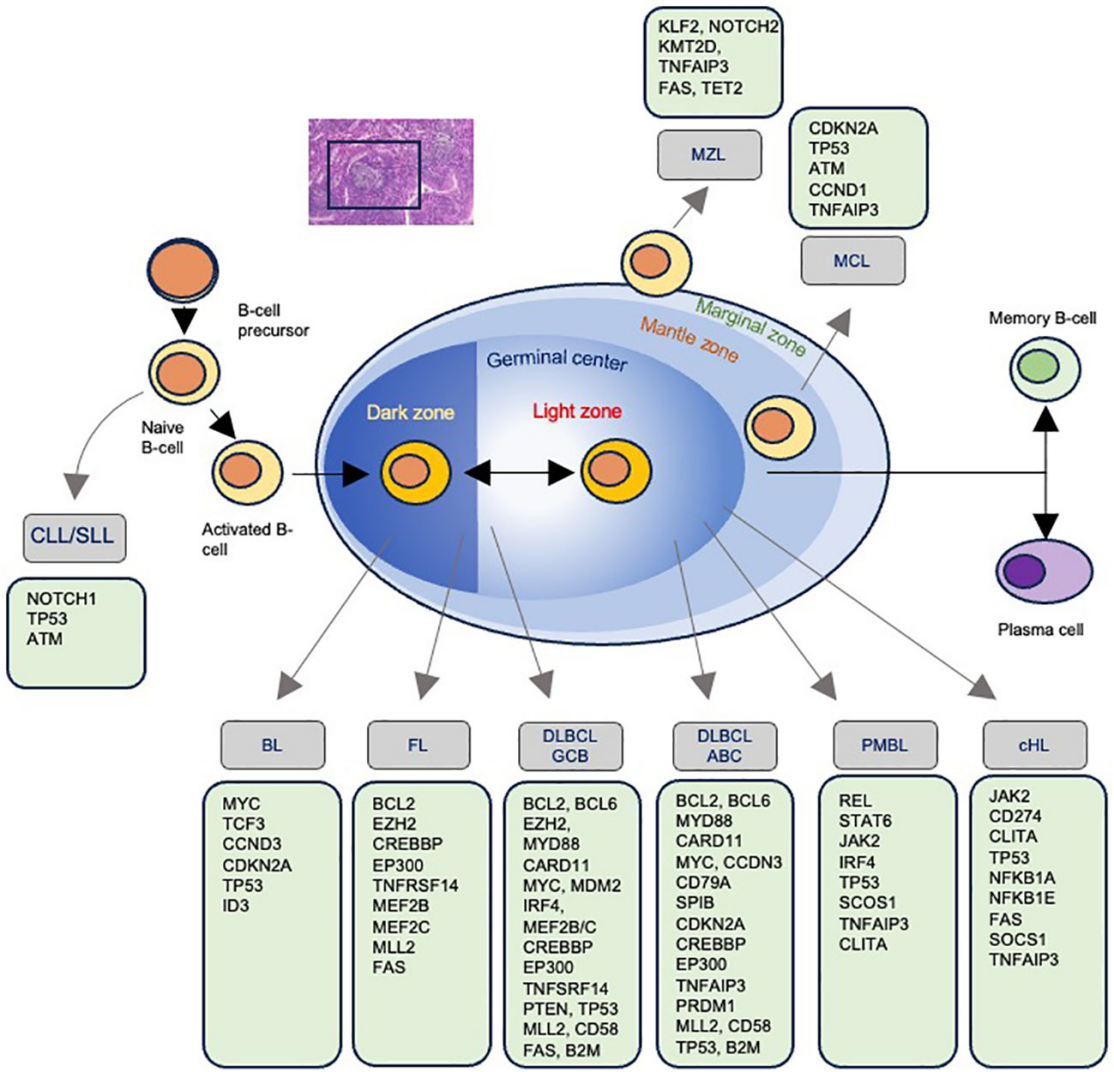
Mutations associated with each type of non-Hodgkin lymphoma. The progression of B-cell maturation and the associated development of lymphoma with the most commonly identified mutations.

Non-Hodgkin lymphoma (NHL) encompasses a diverse group of lymphoid malignancies, each characterized by distinct genetic profiles that may influence radiosensitivity and treatment outcomes (**Figure 4**). Indolent lymphomas, such as follicular lymphoma (FL) and marginal zone lymphoma (MZL), generally exhibit slow disease progression and relatively good responses to treatment, while other lymphomas like MCL, BL and diffuse large B-cell lymphoma (DLBCL) can have more aggressive courses (**Figure 5**). Of note, depending on histology and clinical situation, guideline-supported RT doses acceptable for treatment of NHL range from 4-54 Gy (89). This wide spectrum underscores a critical need to personalize RT dosing.

### Indolent lymphomas

“If he is taking his ease, give him no rest.”

-*The Art of War*, Sun Tzu

Indolent lymphomas are mature B-cell neoplasms that often involve translocations which bring oncogenes in proximity to the immunoglobulin heavy chain (*IGH*) locus at chromosome band 14q32, resulting in overexpression of the oncogene (**Table 3**). For example, FL is commonly defined by t(14;18)(q32;q21) occurring in up to 90% of cases and resulting in overexpression of *BCL2* by placing it under control of the *IGH* promoter. Extranodal marginal zone lymphomas (ENMZL) often arise in the gastrointestinal tract, salivary glands, and other extranodal sites. ENMZLs often have rearrangement involving the *MALT1* gene, such as t(11;18)(q21;q21), t(14;18)(q32;q21), and t(1;14)(p22;q32), which result in the activation of the NF-κB pathway (97). Chromosomal translocations involving the *BIRC3* gene, such as t(1;14)(p22;q32), have also been implicated in MALT lymphomas of the intestine and lung (98). *BIRC3* (also known as *cIAP2*) is an inhibitor of apoptosis, and its dysregulation can contribute to radioresistance by preventing radiation-induced cell death (99). In ENMZLs of the GI system, *BCL10*-*IGH* translocation t(1;14) is a driving feature. In ocular ENMZLs, t(3;14)(p14.1;q32) resulting in *FOXP1*-*IGH* can occur. Other indolent lymphomas, such as nodal MZL and lymphoplasmacytic lymphoma, can also harbor distinct genetic alterations such as *MYD88* and *L265P* mutations, implicated in promoting survival through constitutive NF-κB activation (100). Understanding the genetic landscape of indolent lymphomas provides critical insights into their biology and may inform personalized therapeutic approaches, including the potential for targeted radiosensitization strategies.

**Table 3:** Summary of indolent non-Hodgkin lymphoma and associated genetic aberration (90–96).

Lymphoma type	Known subtypes and prevalence	Major mutated genes and prevalence	Known CNAs	Known translocations	Differentially expressed genes (RNA/Protein)
Follicular Lymphoma (FL)	Grade 1-2: 60-70%, Grade 3A: 15-20%, Grade 3B: 10-15%, Pediatric: <5%, Inguinal type: ~10%, BCL2 translocation negative: ~5-10%	*KMT2D* (60-70%), *CREBBP* (60%), *EZH2* (20%), *BCL2* (85-90%)	1p36 loss, 6q deletion	t(14;18)(q32;q21) - *BCL2*	BCL2 (IHC/FISH), MUM1 (IHC), CD10 (IHC), BIN2, TNFRSF13B, CD69, SLP1, C9ORF52, TNFSRF25, STAT4, IL7R, LEF1, GZMB, BCL2, C4A)
Marginal Zone Lymphoma (MZL)	Nodal: 10%, Splenic: 30-40%, Extranodal: 50-60%	*KMT2D* (30%),*NOTCH2* (20-25%), *KLF2* (10-15%), *PTPRD* (15%), *BIRC3* (5-10%)	7q deletion, 17p deletion	t(11;18)(q21;q21) - *MALT1*, t(14;18)(q32;q21) - *BCL2*	BCL2 (IHC), MALT1 (FISH), BCL6 (IHC), MNDA, TRAF4, CD82, ACI, TNFRSF14, TGFB1
Chronic Lymphocytic Leukemia (CLL)	IGHV-mutated: 50-60%, IGHV-unmutated: 40-50%	*TP53* (10-15%), *ATM* (15-20%), *SF3B1* (10-15%)	13q deletion, 11q deletion, 17p deletion	t(11;14)(q13;q32) - *CCND1*, t(14;19)(q32;q13.3) - *BCL3*	ZAP-70 (IHC), CD38 (IHC), BCL2 (IHC); BANK, CD40L1, ICOS, CRBN, CD19, CD5

### Follicular lymphoma

In FL, genetic mutations occur in epigenetic regulators such as *KMT2D* or *CREBBP* in roughly two-thirds of patients (101). Given the high frequency of epigenetic dysregulation, epigenetic targets are hypothesized to be cancer-driving and involve cell-cycle regulation. If a clear genetic phenotype is identified, it is possible that there may be an observable and exploitable relationship with radiation sensitivity (102). Although no specific mutations have been identified that are directly associated with radiation sensitivity per se, there are already some examples of incorporating genetic information into tools to guide clinical decision making with respect to chemoimmunotherapy. For example, the m7-FLIPI (Follicular Lymphoma International Prognostic Index) score combines clinical factors with genetic mutations in *EZH2*, *ARID1A*, *MEF2B*, *EP300*, *FOXO1*, *CREBBP*, and *CARD11*, providing a more nuanced risk stratification that guides therapeutic decisions and identifies patients who may benefit from more aggressive treatment strategies (103); of note, the utility of m7-FLIPL for prediction of response to chemotherapy is inconsistent (104, 105). A study of the m7-FLIPI on patients from the GALLIUM trial, a clinical study which evaluated rituximab *vs.* obinutuzumab frontline treatment (106), found that *EZH2* mutations were associated with more benefit from cyclophosphamide, adriamycin, vincristine and prednisone (CHOP)/cyclophosphamide, vincristine and prednisone (CVP) chemotherapy, whereas *EZH2* wild-type patients had superior outcomes with bendamustine-based regimens (107).

More recently, genomically distinct subtypes of FL have been identified with different, often more favorable, treatment response (108). Translocation t(14;18) negative FL is noted to arise predominantly in inguinopelvic sites, is enriched for *STAT6* and *CREBBP* mutations, and usually has a good response to chemotherapy and RT (109). Additionally, pediatric-type FL, which occurs in young patients and adolescents, is recognized for its excellent prognosis and high responsiveness to treatment with roughly 50% of cases harbor mutation of *IRF8* and frequent *MAPK* mutations with a surprising absence of epigenetic modifier mutations (110). This subtype is often localized and exhibits a low-grade histology, resulting in long-term remission with standard treatment protocols (111).

### Marginal zone lymphomas

In MZL, most ENMZLs show trisomy of chromosomes 3 and 18. The t(11;18)(q21;q21) translocation resulting an *API2*–*MLT*/*MALT1* is associated with resistance to antibiotic therapy in gastric MALT lymphoma, suggesting a more advanced and therapy-resistant disease (51). In lung cancer cell lines, *MALT1* loss is associated with increased radiosensitivity (112). In *MALT1* rearrangement-negative gastric MALTs, *TRAF3*, *TNFAIP3*, and *NOTCH1* are commonly altered (113). Targeted mutations of *TNFAIP3* are seen in ocular adnexal MALT, possibly associated with more DDR errors and radiosensitivity (114); whereas salivary MALTs have mutations in *TBL1XR1* and *GPR34*, which have been implicated in lung cancer radiation resistance (115). In a study of ocular ENMZL patients using whole genome and targeted sequencing, *JAK3* mutation occurred in 11% of cases and was associated with reduced PFS after chemotherapy relative to wild-type (116). Studies also showed deletions of *TNFAIP3*, and amplifications *of NOTCH* targets and the *CEBP* transcription factor family (116). Mutations of *TBL1XR1* leads to increased NCoR degradation and activation of NF-kB and JUN signaling pathways (117). Lastly, *MYD88* mutations in ocular ENMZL have been associated with inferior disease-free survival after chemotherapy (DFS) (118).

### Aggressive lymphomas

“If your opponent is in superior strength, evade him.”

-*The Art of War*, Sun Tzu

Aggressive lymphomas encompass a subset of NHLs characterized by increased proliferation, more genetic complexity, and higher clinical acuity than indolent lymphomas. Subtypes such as MCL, DLBCL, and BL can exhibit brisk disease progression and are often driven by distinct genetic mutations or chromosomal rearrangements that influence both prognosis and treatment responsiveness (**Table 4**). Despite their aggressive nature, these malignancies can be highly responsive to therapy, especially when their specific molecular features are targeted.

**Table 4:** Summary of aggressive non-Hodgkin lymphoma and associated genetic aberrations (92, 119–123).

Lymphoma type	Known subtypes and prevalence	Major mutated genes and prevalence	Known CNAs	Known translocations	Differentially expressed genes (RNA/Protein)
Diffuse Large B-Cell Lymphoma (DLBCL)	GCB: 40-50%, ABC: 30-40%, Double-Hit: 5-10%, Triple-Hit: 2-3%	*BCL2* (30-40%), *BCL6* (20-30%), *MYC* (10-15%)	3q gain, 18q21 gain, 17p loss	t(14;18)(q32;q21) - *BCL2*, t(3;14)(q27;q32) - *BCL6*, t(8;14)(q24;q32) - *MYC*	BCL2 (IHC/FISH), BCL6 (IHC), MYC (IHC); CD68, BAFF, CD163, KI67, S1PR2
Mantle Cell Lymphoma (MCL)	Classic: 70-80%, Blastoid: 10-15%, Pleomorphic: 5-10%	*CCND1* (95-100%), *TP53* (20-30%), *ATM* (40-75%), *SP140* (10%), *NSD2* (10%)	11q deletion, 13q deletion, 17p loss	t(11;14)(q13;q32) - *CCND1*	Cyclin D1 (IHC), SOX11 (IHC), BCL2 (IHC), RNGTT, HDGFRP3, FARP1, CSNK1E, SETMAR, HMGB3, LGALS3BP, PON2, CDK2AP1, DBN1, CNR1, CNN3, SOX11
Burkitt’s Lymphoma (BL)	Endemic: 40-50%, Sporadic: 30-40%, Immunodeficiency-associated: 10-20%	*MYC* (90-95%), *ID3* (30-40%), *TCF3* (20-30%)	13q deletion, 1q gain, 17p loss	t(8;14)(q24;q32) - *MYC*, t(2;8)(p12;q24) - *MYC*, t(8;22)(q24;q11) - *MYC*	MYC (IHC/FISH), BCL6 (IHC), CD10 (IHC)

### Mantle cell lymphoma

MCL is a subtype of NHL characterized by the presence of characteristic rearrangement t(11;14) involving cyclin D1 (*CCND1*), a cell cycle signaling factor. Cyclin D1 can form a complex with CDK4 or CDK6, both of which are overexpressed in MCL (124). The downregulation of specific microRNAs has been linked to CDK6 upregulation and poorer survival (125). Studies have shown that there are also frequent mutations in the *TP53* and *ATM* genes, which have implications for therapeutic sensitivity. In MCL, mutations in *TP53* are associated with poor prognosis and resistance to conventional therapies (126). *TP53* mutation is associated with MCL blastoid morphology, and its presence informs chemotherapy decision making. *ATM* is mutated in roughly 70% of MCL, and since they are thought to result in defective DNA repair mechanisms, they implicate *ATM* mutation as a likely MCL defining event. The high frequency of *ATM* mutations make it ideal for further study as a clinical biomarker (127). At present, neither *TP53* nor *ATM* alteration are used routinely to guide RT utilization; however, MCL can be extremely sensitive to very low dose radiation (128), and therefore, a relationship between MCL’s altered DDR and radiosensitivity may exist.

Another interesting finding is that MCL has a relatively high degree of CNA compared to other lymphomas, perhaps attributed to mechanisms allowing a bypass of the normal cell cycle checks through cyclin D1/CDK4 (129). CNA in MCL has also been linked to altered *MAP2* and *MAP6*, microtubule genes that could contribute to genomic instability (130). From WES of MCL, recurrent mutations were identified including *WHSC1*, *RB1*, *POT1*, and *SMARCA4 (* 131 *)*. Another study noted 4 mutational signatures in MCL with different overall survival probabilities: mutated *IGH* variable, *CCND1* mutation, amplified 11q13, and active B cell receptor signaling (122). The *CCND1* signature was also associated with del(11q), *ATM* mutations, and upregulation of NF-κB and DNA repair pathways (122). Understanding these genetic mutations may allow for more precise decision making with respect to radiation doses, potentially improving patient outcomes by enhancing the therapeutic ratio.

### Burkitt lymphoma

BL is a highly aggressive form of NHL characterized by the rapid growth of malignant B-cells. There are three distinct clinical subtypes: sporadic BL, endemic BL predominantly in sub-Saharan Africa, and immunodeficiency-associated BL. Genetic studies have demonstrated both common mutations across subtypes (e.g., mutations in *ID3*), and distinct alterations for each subtype (132). BL is most commonly associated with translocation t(8;14)(q24;q32), which juxtaposes the *MYC* gene with *IGH* locus (121). This translocation leads to the overexpression of the MYC oncogene, a critical regulator of cell proliferation, apoptosis, and metabolism. The aberrant expression of *MYC* drives the uncontrolled growth and rapid proliferation of lymphoma cells, contributing to BL’s aggressive clinical behavior. The translocation involving *MYC* can also occur with other immunoglobulin loci, such as t(2;8)(p12;q24) involving the kappa light chain or t(8;22)(q24;q11) involving the lambda light chain, although these are less common. *MYC* dysregulation is a key driver of oncogenesis in BL, and its detection is critical for diagnosis and therapeutic decision making. Studies have shown that MYC overexpression is associated with poor prognosis and resistance to conventional therapies, making it a target of interest for novel therapeutic approaches (133). Sporadic and immunodeficiency-associated BLs were shown to be genetically similar with mutations in *TCF3*, *CCND3*, and *SMARCA4 (* 134 *)*; whereas, endemic BL has more frequent mutations in *BCL7A* and *BCL6* and fewer genetic alterations in *DNMT1*, *SNTB2*, and *CTCF* (132).

### Diffuse large B-cell lymphoma

DLBCL is the most common form of NHL and is characterized by significant genetic and phenotypic heterogeneity, which influences treatment response and prognosis. DLBCL can be classified into two major phenotypes based on gene expression profiles: activated B-cell-like (ABC) and germinal center B-cell-like (GCB), with distinct molecular characteristics and clinical behaviors. ABC DLBCL is often associated with poorer prognosis and resistance to standard therapies, including radiation, due to constitutive activation of the NF-κB pathway (135). In contrast, GCB DLBCL generally has a better prognosis and is more responsive to chemotherapy (136). Despite efforts to classify the cell of origin of DLBCL, precision oncology based on these subtypes has not consistently translated into clinical benefit (137), although upfront Polatuzumab was recently demonstrated to have more benefit for ABC DLBCL than GCB DLBCL (138, 139). Rearrangements, mutations, and overexpression of *BCL2*, *BCL6*, and *MYC* are critical genetic alterations in DLBCL that further influence treatment outcomes. *BCL2* rearrangements are more common in GCB DLBCL and are associated with resistance to apoptosis, which can impact the effectiveness of radiation therapy (140). *BCL6* rearrangements, which are found in both ABC and GCB subtypes, can inhibit differentiation and promote survival of lymphoma cells, potentially affecting their radiosensitivity (141). *MYC* rearrangements, often associated with a more aggressive disease course, lead to overexpression of the MYC protein, driving rapid cell proliferation and metabolic activity. The co-occurrence of *MYC* rearrangements with *BCL2* and/or *BCL6* rearrangements, known previously as “double-hit” or “triple-hit” lymphomas, now known as High-Grade B-Cell Lymphoma (HGBCL), is associated with very poor prognosis and resistance to conventional treatments (142). MYC-positive cells are shown to have more oxidative stress and replication errors which lead to DNA damage and genomic instability (143). DDR activation in DLBCL correlates with *MYC* expression and predicts poor prognosis (144). In fact, inhibition of *ATR*-*CHK1*/*2* mediated DDR was linked to chemotherapy resistance in *MYC*-positive DLBCL (145).

Early WES studies of DLBCL, showed recurrent mutations in *MYD88*, *CARD11*, *EZH2*, and *CREBBP*, which were known to be altered in DLBCL and somatic mutations in novel genes like *MEF2B*, *MLL2*, *BTG1*, *GNA13*, *ACTB*, *P2RY8*, *PCLO*, and *TNFRSF14 (* 146 *)*. Later studies compared ABC and GCB subtypes to show GCB type preferentially mutated in *EZH2*, *SGK1*, *GNA13*, *SOCS1*, *STAT6*, and *TNFRSF14*, and ABC type biased toward mutations *ETV6*, *MYD88*, *PIM1*, and *TBL1XR1 (* 147 *)*. Loss of *CDKN2A* is associated shorter survival after Rituximab-CHOP through dysregulation of the RB/E2F pathway, activation of cellular metabolism, and decreased immune and inflammatory responses (148). Shipp et al. identified five DLBCL genetic phenotypes including transformed from indolent lymphoma, two subsets of GCB-DLBCLs and group with biallelic inactivation of *TP53*, *CDKN2A* loss, and associated genomic instability (142). The LymphGen classification further refines the molecular categorization of DLBCL into distinct genetic subgroups with specific phenotypic and clinical characteristics (149). This classification includes subtypes MCD (*MYD88L265P* and *CD79B* mutations), BN2 (*BCL6* fusions and *NOTCH2* mutations), N1 (*NOTCH1* mutations), EZB (*EZH2* mutations and *BCL2* translocations), ST2 (PI3K signaling and JAK2 signaling), A53 (low p53 target genes), and TP53Mut (p53 signaling dysregulation, immune deficiency, and PI3K activation), each associated with different therapeutic responses and outcomes (150). Lastly, primary central nervous system lymphoma (PCNSL) is an aggressive large B-cell lymphoma that also displays *CDKN2A* loss and mutations in *MYD88*, *CD79B*, and *TBL1XR1*, thus with potentially definable genetic subtypes (151). Understanding these genetic and phenotypic subtypes may allow for more precise tailoring of radiation therapy protocols. For instance, patients with *MYC*, *BCL2*, and/or *BCL6* rearrangements may benefit from more aggressive treatment regimens or novel therapeutic combinations that enhance radiosensitivity and overcome resistance mechanisms.

### T-cell lymphomas

T-cell lymphomas are a heterogeneous group of malignancies characterized by various genetic alterations that influence their response to treatments. Among the key genetic mutations associated with T-cell lymphomas are those in the *TP53* and anaplastic lymphoma kinase (*ALK*) genes, which significantly impact disease behavior and treatment outcomes (152). Early genetic studies exploring mutations in *ATM* and *TP53* showed that mutant mice developed T-cell lymphoma with mice having both genes knocked out displaying resistance to radiation (153). In T-cell lymphomas, *TP53* mutations can lead to impaired apoptosis and increased survival of malignant cells despite radiation-induced DNA damage, suggesting a potential for reduced radiation sensitivity (154). Conversely, *ALK* mutations or translocations, particularly in *ALK*-positive anaplastic large cell lymphoma (ALCL), are associated with a distinct clinical and biological profile (155). *ALK*-positive T-cell lymphomas generally exhibit better responses to chemotherapy and RT compared to *ALK*-negative variants, likely due to the oncogenic driver role of *ALK* mutations that render the cells more susceptible to targeted treatments, though no clear relationship with radiation is known (156). The potential to understand variability in radiation sensitivity based on genetic alterations underscores the importance of molecular profiling in T-cell lymphomas to optimize treatment strategies and improve patient outcomes.

### Plasma cell neoplasms

Plasma cell neoplasms like solitary plasmacytoma and multiple myeloma (MM) have also shown associations with mutations in the *TP53* and *ATM* genes. Initial reports showed roughly a third of extramedullary MM reported t(4;14), deletion of 13q the *RB1* locus, and deletion 17p the *TP53* locus. Recently, WES of extramedullary MM showed most patients had 1q21 amplification including the *CKS1B* gene and at least one mutated gene in the MAPK signaling pathway, with *KRAS* as the most frequently mutated gene (157). *NBS1* mutations in myeloma may contribute to carcinogenesis and may be targetable (158). Additionally, MM polymorphisms in DNA repair genes such as *XRCC1* have been identified and could potentially influence radiosensitivity and treatment responses across myeloma and various lymphoid malignancies (159).

### Transformed hematologic malignancies

While chronic lymphocytic leukemia (CLL) is not thought to have a large amount of genetic dysregulation, Richter’s syndrome (RS), a transformation to aggressive disease, has recently been characterized through WGS with some recurrent mutations observed (160). Mutations were found in the DDR pathway, but also other genes like *PTPRD* and *TRAF3*. Immune genes were also implicated including *BTG2*, *CXCR4*, *NFATC1*, *PAX5*, and *NOTCH*. Transformed FL may be associated with *CDKN2A*/*B* deletions which are also associated with inferior PFS and OS after Rituximab (161).

## Discussion

### Radiogenomics in hematologic malignancies

“The rule is, not to besiege walled cities if it can possibly be avoided”

-*The Art of War*, Sun Tzu

While successful efforts in non-hematologic malignancies have linked radiation sensitivity to genomic classifiers for solid tumors, implicating genes like *KEAP1* and *CTNNB1* and pathways such as ROS reduction and cell-cycle deregulation, these studies have often explicitly omitted hematologic malignancies (162). In the absence of clear molecular markers of radiosensitivity, radiation oncologists determine radiation candidates, targets, and doses, based on factors including existing retrospective and prospective trials, lymphoma aggressiveness, disease burden, disease location, and chemotherapy response, though practice patterns may vary. As different hematologic cancer subtypes exhibit both distinct and common molecular traits that influence disease behavior and treatment response, we are now in an era of where integrating genomic information into treatment decision-making is possible. The prospect of incorporating genetic information into a radiation treatment plans has given rise to the exciting, emerging field of radiogenomics. Radiogenomics promises to identify genetic markers that can predict RT toxicity (163), and better predict and prognosticate RT efficacy (164). By leveraging genomic data, oncologists can already tailor treatment plans to individual patients in medical oncology, enhancing the precision and efficacy of chemoimmunotherapy, and similar techniques should exist in radiation oncology.

Genomic tools may offer a powerful method to help determine which patients are most likely to respond well to RT. While chemotherapy alone can effectively control malignancies, there are specific situations where RT after chemotherapy may be necessary, such as a bridging strategy before CAR T-cell therapy, cytoreduction prior to transplant, or consolidation therapy for high-risk disease. Identifying specific genomic markers linked to radiosensitivity can guide these decisions, particularly in cases like MCL and HL, where combined modality therapy may offer advantages, potentially reducing the need for extensive chemotherapy. In the pre-rituximab era, efforts like SWOG 8736 established that fewer cycles of chemotherapy combined with radiation were superior in treating DLBCL compared to more cycles of chemotherapy alone (165). Future efforts are needed to determine which genetic markers reliably identify patients who can tolerate reduced-intensity chemotherapy when additional cycles are substituted for radiotherapy.

Currently, it is unknown whether signals of radiosensitivity exist in hematologic malignancies. Recently, in an abstract, CREBBP alterations in FL were shown to predict response to very low dose RT, offering a significant advancement in RT personalization (166). However, in a study of higher RT doses in early-stage FL, no gene alterations were associated with outcomes (167). Similarly, Ma et al. explored genomic correlates of radiosensitivity in DLBCL, focusing on the LymphGen classification (168). Studies like these could stratify patients from the outset based on whether certain subtypes will likely benefit from lower-dose regimens. For example, *ATM*-mutant MCL could be preferentially treated with 4 Gy in an adaptive RT approach, potentially minimizing RT exposure for specific MCL patients. Additionally, genomic profiling opens opportunities for novel treatment combinations with lower RT doses. For example, incorporating EZH2 inhibitors with low-dose RT could be explored for specific LymphGen subtypes in relapsed/refractory DLBCL. Furthermore, radiopharmaceuticals could be utilized for patients with systemic disease predicted to have high RT sensitivity based on their genomic profile. However, challenges in accessing radiopharmaceutical agents and regulatory hurdles limit their use. Personalized genomic profiling may justify broader adoption of radiopharmaceuticals, which could also be combined with immune checkpoint inhibitors to unlock new treatment avenues for systemic lymphomas. By moving beyond standard approaches, these novel strategies have the potential to improve outcomes in patients with challenging lymphoma cases.

Bulky tumors, which are often less sensitive to radiation, might benefit from the addition of radiosensitizers based on specific genomic vulnerabilities. Conversely, identifying patients at lower risk of long-term RT toxicities—through germline or somatic mutations affecting DNA repair pathways—could lead to better-informed decisions about dose and modality, allowing oncologists to balance efficacy and safety. These personalized approaches align with the overarching goal of optimizing radiation oncology through genomics, ensuring that each patient receives the most appropriate treatment for their unique cancer profile.

Future directions in this field are promising, with ongoing research aimed at refining genomic profiling techniques and expanding our understanding of the molecular mechanisms underlying radiosensitivity. Studies are needed to investigate the significance of mutations in commonly mutated cell cycle-regulating genes like *TP53*, *MYC*, *BCL2*, and *BCL6* and DDR genes like *ATM*. Additionally, associations between lymphoma and epigenetic modifiers offer clinically useful biomarkers given their high frequency and connection to disease development. As genomic technologies continue to evolve, they hold the potential to transform radiation oncology, making personalized treatment a standard practice. Collaborative efforts like the Radiogenomics Consortium will be essential in overcoming current obstacles and realizing the full potential of genomics in optimizing radiation therapy for lymphoma patients (169).

Recognizing the transformative potential of genomics in radiation oncology within the framework of precision medicine, the American Society for Radiation Oncology, American Association of Physicists in Medicine, and the National Cancer Institute convened a Precision Medicine in Radiation Oncology Workshop (170). The group emphasized the urgent need to develop and validate genomic markers for predicting radiosensitivity in tumors and normal tissues, with a focus on creating polygenic risk scores. They also called for deeper exploration of molecular mechanisms underlying radiosensitivity and resistance, including hypoxia, HPV status, and alterations in DNA damage response pathways, while investigating synergies between radiation therapy and systemic treatments like immune checkpoint inhibitors and targeted molecular therapies.

To support these goals, the workshop advocated for multi-institutional consortia such as ORIEN to pool resources for genomic research (171), cooperative group trials that integrate genomics into radiation therapy protocols, and the expansion of genomic repositories to include detailed radiation therapy data such as dose-volume histograms and locoregional recurrence rates. The group stressed the importance of designing randomized trials to assess the benefits of genomically personalized radiation therapy, including selective dose escalation and chemoradiotherapy combinations tailored to genetic profiles. Practical recommendations included enhancing radiation oncologists’ genomic literacy through residency curricula, symposia at national meetings, and dedicated study sections within funding agencies like the NIH to prioritize precision radiation oncology research. Lastly, the group highlighted the importance of fostering academic-industry collaborations to advance radiation-guided precision oncology platforms, drawing inspiration from the pharmaceutical industry’s success in integrating precision medicine.

### Challenges in translating genetic research into clinical applications

“He will win who knows when to fight and when not to fight.”

-*The Art of War*, Sun Tzu

Translating genetic research into the clinic poses numerous challenges, largely due to the complexity and variability of genetic data. The transition from candidate gene studies to GWAS necessitates robust validation and reproducibility (172), and large, well-designed studies to confirm associations before clinical use (173). Ethical, legal, and social implications also play a significant role (174), as incorporating genetic information into cancer care issues of consent, privacy, and potential discrimination. Integrating genetic data into clinical workflows presents practical challenges, requiring comprehensive clinical decision support systems, timely availability during clinical decision-making, and extensive training for healthcare providers for interpretation (70). Technical and methodological barriers, such as the need for standardized protocols and the complexity of polygenic models, further complicate the clinical translation of genetic research (172). Economic and resource constraints are additional obstacles resulting in financial and infrastructural requirements for implementing genetic testing and genomic data analysis in clinical practice (175). Moreover, the acceptance of genetic testing by both patients and physicians remains a significant barrier, and education is needed to facilitate the adoption of genetic research in clinical settings (176). Regulatory and policy issues also need to be addressed, highlighting the necessity for clear guidelines and policies to support the integration of genetic research into clinical practice (177). Future research should focus on addressing these challenges, developing more precise and comprehensive genomic profiles, and validating predictive models in large, diverse patient populations.

## Conclusion

**“**Ponder and deliberate before you make a move.”

-*The Art of War*, Sun Tzu

The intersection of genetic research and radiation therapy in hematologic malignancies represents a promising frontier for personalized medicine. Over the years, substantial progress has been made in understanding the molecular underpinnings of radiosensitivity through the study of key genes involved in DNA repair and cell cycle regulation. Syndromes such as Ataxia-Telangiectasia, Nijmegen Breakage Syndrome, and Li-Fraumeni Syndrome have provided critical insights into the role of *ATM*, *NBS1*, and *TP53* mutations in radiation response. Additionally, advances in genomic profiling have revealed complex mutational landscapes in various types of non-Hodgkin lymphomas, including FL, MCL, and MALT. These discoveries have paved the way for the development of predictive tools which combines clinical and genetic information to guide therapeutic decisions and optimize treatment outcomes.

The war on cancer needs to be fought with smarter, more deliberate tactics, and future research should focus on validating genetic markers of radiosensitivity through large, well-designed clinical trials and exploring novel therapeutic combinations to overcome resistance mechanisms. The emerging field of radiogenomics holds great potential to enhance the precision of radiation therapy, minimize adverse effects, and improve patient outcomes. By bridging the gap between genetic research and clinical application, we can move closer to realizing the full potential of personalized RT in the treatment of hematologic malignancies.
